# User acceptance of a picture archiving and communication system (PACS) in a Saudi Arabian hospital radiology department

**DOI:** 10.1186/1472-6947-12-44

**Published:** 2012-05-28

**Authors:** Bakheet Aldosari

**Affiliations:** 1 Department of Health Informatics, King Saud bin Abdulaziz University for Health Sciences, Mail Code: 2350, PO Box 22490, Riyadh, 11426, Kingdom of Saudi Arabia

## Abstract

**Background:**

Compared with the increasingly widespread use of picture archiving and communication systems (PACSs), knowledge concerning users’ acceptance of such systems is limited. Knowledge of acceptance is needed given the large (and growing) financial investment associated with the implementation of PACSs, and because the level of user acceptance influences the degree to which the benefits of the systems for healthcare can be realized.

**Methods:**

A Technology Acceptance Model (TAM) was used to assess the level of acceptance of the host PACS by staff in the radiology department at King Abdulaziz Medical City (KAMC), Riyadh, Saudi Arabia. A questionnaire survey of 89 PACS users was employed to obtain data regarding user characteristics, perceived usefulness (PU) (6 items), perceived ease of use (PEU) (4 items), a change construct (4 items), and a behavior (acceptance) construct (9 items). Respondents graded each item in each construct using five-point likert scales.

**Results:**

Surveyed users reported high levels of PU (4.33/5), PEU (4.15/5), change (4.26/5), and acceptance (3.86/5). The three constructs of PU, PEU, and change explained 41 % of the variation in PACS user acceptance. PU was the most important predictor, explaining 38 % of the variation on its own. The most important single item in the explanatory constructs was that users found PACS to have improved the quality of their work in providing better patient care. Technologists had lower acceptance ratings than did clinicians/radiologists, but no influence on acceptance level was found due to gender, age, or length of experience using the PACS. Although not directly measured, there appeared to be no cultural influence on either the level of acceptance or its determinants.

**Conclusions:**

User acceptance must be considered when an organization implements a PACS, in order to enhance its successful adoption. Health organizations should adopt a PACS that offers all required functions and which is likely to generate high PU on the part of its users, rather than a system that is easy to use. Training/familiarization programs should aim at establishing high levels of PU in all users, particularly technologists. Health organizations are advised to measure all the factors that influence the acceptance of a PACS by their staff, in order to optimize the productivity of the system and realize the potential benefits to the greatest extent possible.

## Background

New technologies are frequently being adopted by healthcare organizations in order to try to improve the quality and efficiency of healthcare [1,2]. The picture archiving and communication system (PACS) is an example of such a technology. A PACS is a medical image management information system which manages medical images and integrates equipment through a network. Such a system allows digital images to be stored in a database and retrieved using a file management server, to be transmitted using computer networks, to be displayed at various resolutions to users with different requirements, and to be analyzed and processed as a reference for medical treatment. It is now common for a PACS to be integrated as a module within a wider Radiology Information System (RIS) or Hospital Information System (HIS) [1]. Users of a PACS include technologists, image library personnel, radiologists, physicians/clinicians, and nurses.

The tangible benefits of a PACS (cf. film-based systems) are well established and numerous, and include ([1-8]): improving the operational efficiency and productivity of the medical image service; allowing the availability of images anytime and anywhere; reducing waiting times for image retrieval and turn-around times of clinical reports; attaching scans to patients’ electronic health records; scheduling the use of radiology equipment more efficiently; facilitating long-distance consultations; providing auxiliary tools to support image diagnosis; and improving hospital workflow, with subsequent benefits for patient care. Intangible benefits include increased satisfaction with the service on the part of radiology staff and referring physicians, and increased satisfaction of patients with their care [5].

Overall, the advent of PACSs has resulted in a dramatic simplification of image management for host health organizations. However, PACS implementation and adoption, like many other healthcare IT implementations [2], represents a major change in a healthcare organization and has proved to be a substantial challenge to many such healthcare organizations [3,9]. Managing change to overcome clinician resistance and increase acceptance may pose a significant obstacle to any successful IT adoption, including that of a PACS [3,10,11]. The best and most expensive IT system will be ineffective if it is resisted by its users. Given the anticipated expanded application of IT systems in healthcare and the increasingly large financial and other resources being allocated to them [1,2,11], then human factors, including user acceptance, become even more important. Or, as Ward et al. [11] put it, “the factors which influence staff attitudes towards [the IT systems] become increasingly significant if the investment is to be worthwhile” (p.93). In the case of PACSs, the global market is forecast to increase from $2.8 billion in 2012 to $5.4 billion in 2017 [12]. The Middle-East market for PACSs-RISs was estimated at $86 million in 2009 and could grow to $140 million by 2014 [13]. The number of hospitals with PACSs or RISs in the Middle-East was 984 in 2010 and is set to increase to 1680 by 2014. In 2009, one-third of PACS installations in the region took place in Saudi Arabia.

Given the levels of investment associated with the adoption of PACSs, and the need to gain as much benefit from the systems as possible, the importance of user acceptance to the success of PACS implementations in health organizations is clear. Various studies have investigated the determinants of behavioral intention (acceptance) with respect to IT implementation [11,14-16]. Several different types of model have been developed to study IT acceptance, including the Technology Acceptance Model (TAM), the Diffusion of Innovation model, the Information Systems Success model, the Social-Cognitive Theory model, and the Task-Technology Fit model [17-20]. This study uses a variant of the TAM of Davis [21] to assess the behavior (acceptance) of staff with respect to the host PACS in King Abdulaziz Medical City (KAMC), a hospital in Riyadh, Saudi Arabia. The TAM (and its variants and extensions) has been shown to be appropriate for evaluating user acceptance behavior toward IT systems, including in the healthcare setting [14,15,17,22]. Holden and Karsh’s review [22] found 16 datasets in more than 20 studies where the TAM (or a variant or extension thereof) has been applied to clinician use of health IT.

Despite the importance of PACSs for healthcare organizations, the large financial investments involved, and the increasingly widespread use of these systems, there have been surprisingly few investigations into users’ acceptance of this healthcare technology. Published studies of aspects of PACS acceptance include those of Pare and Trudel [3], Duyck et al. [6], Hurlen et al. [7], Bramson and Bramson [10], Duyck et al. [23], Pare et al. [24], Pynoo et al. [25], and Duyck et al. [26]. However, of those, only five studies ([6,23-26]) used a model of technology acceptance to quantitatively investigate PACS acceptance, and as three of the studies ([6,23,26]) used the same questionnaire survey database, just three unique databases are represented in the literature prior to this study. The number of studies of PACS acceptance therefore is remarkably small compared with the widespread, and increasing, use of the system. This study additionally represents the first investigation of PACS acceptance using a TAM (or variant/extension thereof) to be performed in a hospital in an Arab country.

### The technology acceptance model (TAM)

**Figure 1:**
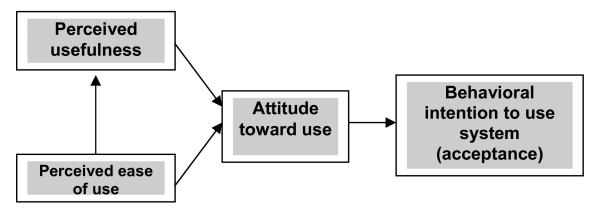
The technology acceptance model (TAM) of Davis [21].

Derived in its original form from the theory of reasoned action (TRA) [27], the TAM explains how users accept and use a technology (Figure 1). Developed by Davis [21], the model regards two acceptance measures as the primary determinants of behavioral intention to use technology: (1) Perceived usefulness (PU), defined as "the degree to which a person believes that using a particular system would enhance his or her job performance”; and (2) Perceived ease of use (PEU), defined as "the degree to which a person believes that using a particular system would be free from effort" ([21], p.985). The model proposes that PEU has a causal effect on PU, and that each of these has an influence on the user’s attitude towards use; both PU and attitude toward use influence behavioral intention (acceptance), which in turn influences usage [17].

The original TAM was extended (to create TAM2) [28] by adding some additional drivers of PU and PEU, including theoretical constructs of social influence processes and cognitive instrumental processes. There have also been attempts to enhance the explanatory power of the TAM [15,19] by adding antecedent, mediating, and moderating variables to explain acceptance behavior in different settings. Lee et al. [16], in their review of the TAM, found that more than 20 different factors have been introduced into various versions of the model, including constructs representing education level, computer expertise, voluntariness, fit between user and system design features, management support, and socio-demographic variables. A later extension of the TAM was formulated by Venkatesh et al. [19] as the Unified Theory of Acceptance and Use of Technology (UTAUT). The UTAUT incorporates four direct determinants of behavioral intention (acceptance), which are performance expectancy (equivalent to PU), effort expectancy (equivalent to PEU), social influence, and facilitating conditions. The model also contains four moderating variables (experience, voluntariness, gender, and age) that have been found to be significant in some studies of technology acceptance.

Specific to the healthcare context, Holden and Karsh [22] found in their overview of healthcare-related TAM studies that almost all such studies had added variables to the basic TAM in an effort to better understand the antecedents of health IT acceptance or usage behavior. These variations have been added “to account for the complexity of healthcare’s socio-technical systems” ([22], p.166). Topics in healthcare have included the acceptance of telemedicine, electronic health records systems, computerized physician order entry systems, mobile healthcare systems, and PACSs [15,22].

### Study objectives

This study uses a variant of the TAM of Davis [21] to assess the acceptance of the host PACS by radiology staff at KAMC, Riyadh, Saudi Arabia. The specific objectives of the study are: 1) To assess radiology user acceptance (behavior) regarding the PACS in the radiology department at KAMC, in Riyadh, Saudi Arabia; 2) To determine the extent to which three constructs (perceived usefulness, perceived ease of use, and change) influence user acceptance; and 3) To determine whether user acceptance is explained by socio-demographic variables.

## Methods

A modified TAM was applied to measure staff acceptance of a PACS in KAMC in Riyadh city, Saudi Arabia. The hospital has a 1,025-bed capacity and hosts various state-of-the-art medical and surgical facilities. The Radiology Department is a busy one; in 2006, the department performed more than 160,000 diagnostic and interventional examinations. The PACS system is a mandatorily used system, and went “live” in January 2005. A survey questionnaire was designed and used to elicit the views of the target population on aspects of the PACS. The target population for the survey comprised 24 consultants, 13 radiologists, 15 residents, 103 technologists, and others who used PACS in their work in the radiology department. Given that some staff were on leave at the time, 120 questionnaires were distributed in May 2008.

**Figure 2:**
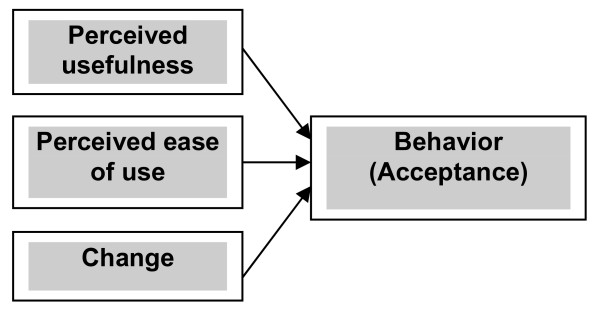
Model framework used in this study.

Part of the questionnaire (detailed further below) measured the four constructs of PU, PEU, change, and behavior (acceptance), and were related structurally as shown in Figure 2. The model used differs from the original TAM by adding a change construct, removing attitude toward use as a variable (as in TAM2), and removing the influence of PEU on PU (as in the UTAUT) (see Figure 1 of Holden and Karsh [22] for a clear exposition of TAM, TAM2, and UTAUT). In the model, PU and PEU were defined as originally proposed by Davis [21] and operationalized according to the recommendations of Davis and other studies (as summarized in Table 1 of Holden and Karsh [22]), with a modified six-item scale for PU and a modified four-item scale for PEU. The change construct was defined as the movement of an organization from its present to a future state, and was operationalized using a four-item scale. The behavioral (acceptance) scale was defined as the adjustment in behavior of a person or group in response to new or modified surroundings, and was operationalized with a nine-item scale.

**Table 1:** Frequency distribution of socio-demographic variables of the questionnaire respondents (n = 89)

**Variable**	**Frequency**	**%**
**Gender:**		
(1) Male	51	57.3
(2) Female	38	42.7
**Total**	**89**	**100**
**Age:**		
(1) 22-30	39	43.8
(2) 31-39	25	28.1
(3) 40-49	15	16.9
(4) ≥ = 50	7	7.9
**Total**	**86**	**96.7**
**Job title:**		
(1) Consultant	12	13.5
(2) Radiologist	5	5.6
(3) Resident	6	6.7
(4)Technologist	64	71.9
(5) Others	2	2.2
**Total**	**89**	**100**
**PACS Experience:**		
(1) ≤ one year	17	19.1
(2) > one year	72	80.9
**Total**	**89**	**100**
**Use of PACS:**		
(1) Always	84	94.4
(2) Frequently	4	4.5
(3) In the past but not now	1	1.1
**Overall**	**89**	**100**

### The instrument

The study instrument (questionnaire) consisted of two parts. Part 'A' included questions about respondent characteristics including age, gender, and job title, and about respondents’ length of experience with PACS and how frequently they used the system in their daily work, following Venkatesh et al. [19] and Duyck et al. [6]. The five variables measured and the response options were: (1) Age (open); (2) Gender (male, female); (3) Job title (consultant, radiologist, resident, technologist, other); (4) PACS experience (≤ 1 year, > 1 year); (5) PACS use frequency (in the past but not now, occasionally, frequently, always).

Part 'B' of the questionnaire included three sections. The first section was a use scale (measuring both PU and PEU) that contained ten statements, with statements 1–6 reflecting perceived usefulness and statements 7–10 reflecting ease of use. The participants graded their responses on each item using a five-point likert scale: strongly disagree = 1, disagree = 2, neither agree/disagree = 3, agree = 4 and strongly agree = 5. The items (statements) were: (1) Using the PACS enables me to accomplish tasks more quickly. (2) Using the PACS has improved the quality of my work in providing better patient care. (3) Using the PACS has increased my productivity. (4) Using the PACS has enhanced my effectiveness on the job. (5) Using the PACS has made my job easier to perform. (6) Using the PACS has given me greater control over my work schedule. (7) Learning to use the PACS has been easy for me. (8) My interaction with the PACS has been clear. (9) My interaction with the PACS has been understandable. (10) It is easy to become skillful at using the PACS.

The second section was a change construct which contained four statements in response to the question “How has the PACS made your job”? Customized five-point likert scale responses were used for the four statements, as follows: (1) More difficult = 1, difficult = 2, neutral = 3, easy = 4, easier = 5. (2) Not interesting = 1, less interesting = 2, neutral = 3, interesting = 4, more interesting = 5. (3) Extremely more stressful = 1, more stressful = 2, neutral = 3, stressful = 4, less stressful = 5. (4) Extremely less pleasant = 1, less pleasant = 2, neutral = 3, pleasant = 4, more pleasant = 5.

The third section was a behavioral (acceptance) construct which contained nine statements. The behavioral construct was used to represent the user’s behavior with respect to the PACS. The participants graded their responses on each item using a five-point likert scale: never = 1, occasionally = 2, fairly frequently = 3, very frequently = 4, always = 5. Five of the statements were “negative” (statements 2, 3, 5, 7, and 9) and were reversed in their scale values. The items (statements) were: (1) Admiring the PACS. (2) Difficulty learning to use the PACS. (3) Complaining about the PACS. (4) A high level of proficiency learning to use the PACS. (5) Lack of cooperation with PACS personnel. (6) Using the PACS appropriately. (7) The PACS slowing work performance. (8) Enjoying working on the PACS. (9) Bypassing the PACS, i.e., using film-based procedures to perform tasks.

After formulating the items of the constructs in accordance with the literature, two steps were followed to increase the content validity of the questionnaire. First, the comments and suggestions of PACS administrators were taken into account. Second, a pilot study was performed on a small sample of consultants, radiologists, residents, and technologists who were using PACS in King Khaled University Hospital in Riyadh, after which minor adjustments were made to some aspects of the questionnaire.

### Data analysis

Of the 120 questionnaires distributed, 89 (74 %) were returned completed. Questionnaire data were analyzed with SPSS (Statistical Package for Social Science). Descriptive analysis used in the study included frequencies, percentages, means, and standard deviations. Inferential analysis included independent and paired sample t-tests, ANOVA, and stepwise multiple regression. The significance level used for the inferential statistics was 0.05. A sample mean approach was used to replace missing values.

## Results

### Questionnaire reliability

The reliability of the questionnaire was measured using Cronbach’s alpha. Values of this measure were 0.88 for the use (PU and PEU) scale, 0.72 for the change construct, and 0.74 for the acceptance construct. Cronbach's alpha should not be less than 0.70 for newly developed instruments [29].

### Respondent characteristics

The age of participants in the study ranged from 22 to 69 years old with an average of 34.5 years. Fewer than half the participants were aged 30 or younger (Table 1). Seventeen (19.1 %) of the participants each had one year or less experience with PACS and 72 participants (80.9 %) each had more than one year experience.

### Use, change, and behavior constructs

With regard to PU, the respondents strongly agreed that the PACS has been a useful tool for practicing their profession (Table 2), with a mean PU rating of 4.33 and standard deviation 0.16. The highest ratings for PU related to task speed, quality of work, job ease, and productivity; the lowest rating was assigned to control over work schedule, although that item still achieved an overall score of more than 4.0. The respondents also rated the PACS highly in terms of ease of use of the system (Table 2), with a mean PEU rating of 4.15.

**Table 2:** Items and scores for the Perceived Usefulness construct, Perceived Ease of Use construct, Change construct, and Behavior construct

**Perceived Usefulness**	**Mean**	**Std Dev.**
1. Using the PACS enables me to accomplish tasks more quickly.	4.42	0.72
2. Using the PACS has improved the quality of my work in providing better patient care.	4.46	0.72
3. Using the PACS has increased my productivity.	4.35	0.74
4. Using the PACS has enhanced my effectiveness on the job.	4.28	0.77
5. Using the PACS has made my job easier to perform.	4.43	0.71
6. Using the PACS has given me greater control over my work schedule.	4.03	0.86
**Overall**	**4.33**	**0.16**
**Perceived Ease of Use**		
1. Learning to use the PACS has been easy for me.	4.26	0.63
2. My interaction with the PACS has been clear.	4.11	0.70
3. My interaction with the PACS has been understandable.	4.02	0.78
4. It is easy to become skillful at using the PACS	.19	0.69
**Overall**	**4.15**	**0.10**
**Change**		
The PACS has made my job…		
More difficult, difficult, neutral, easy, easier.	4.47	0.68
Not interesting, less interesting, neutral, interesting, more interesting.	4.26	0.67
Extremely more stressful, more stressful, neutral, stressful, less stressful.	4.13	1.04
Extremely less pleasant, less pleasant, neutral, pleasant, more pleasant.	4.17	0.74
**Overall**	**4.26**	**0.54**
**Behavior (Acceptance)**		
1. Admiring the PACS.	3.56	1.23
2. Difficulty learning to use the PACS.	4.34	0.78
3. Complaining about the PACS.	3.75	0.93
4. A high level of proficiency for learning to use the PACS.	2.83	1.19
5. Lack of cooperation with PACS personnel	4.22	1.00
6. Using the PACS appropriately.	3.85	1.12
7. The PACS slowing work performance.	3.96	0.95
8. Enjoying working on the PACS.	4.04	1.04
9. Bypassing the PACS, i.e., using film-based procedures to do things.	4.20	1.10
**Overall**	**3.86**	**0.53**

Concerning the change construct, the respondents reported that the PACS has made their job easy (a mean rating of 4.47) (Table 2). They also found that the PACS has made their job interesting (mean rating of 4.25), pleasant (4.16), and less stressful (4.13).

The behavioral construct was the dependent variable used to measure the users’ acceptance levels (Table 2). The overall behavior rating of the PACS in the radiology department in KAMC was 3.86, which suggests that the PACS users showed a fairly high level of acceptance of the system. There were more item scores <4 in the behavior construct (5/9) than in the other constructs (all items in which scored >4). The item “a high level of proficiency for learning to use the PACS” yielded by some margin the lowest individual rating.

Table 3 summarizes the descriptive statistics of the use construct, change construct, and behavioral construct. It seems clear with regard to the PACS that the users showed high levels on both the use construct and the change construct with overall mean ratings of 4.26, and that they also accepted the PACS as indicated by the behavioral scale, although at a slightly lower level (3.86).

**Table 3:** Descriptive statistics for the constructs

	**n**	**Min**	**Max**	**Mean**	**Std Dev.**
Behavior	89	2.83	4.34	3.86	0.53
Change	89	4.13	4.47	4.26	0.54
PU/PEU	89	4.02	4.46	4.26	0.51

### Factors influencing the overall level of acceptance

Stepwise Multiple Regression (SMR) was used to determine the significant variables influencing, PACS acceptance. The results of the SMR (Table 4) reveal that the overall acceptance (behavioral construct) was significantly related to the three constructs (change, PU, and PEU). The most important factor accounting for the variance in overall acceptance was PU (beta = 0.327, p < 0.01). The R^2^ value indicates that this variable accounted for 38 % of the variation in the acceptance level. The second factor was the change construct (beta = 0.253, p < 0.05), which accounted for 4.5 % of the variance in acceptance. The third factor was PEU (beta = 0.227, and p < 0.05), which accounted for 3.5 % of the variance in overall acceptance. Therefore, each construct of PU, change, and PEU had a significant influence on users’ PACS acceptance, but PU was the strongest determinant.

**Table 4:** Results of stepwise multiple regression of behavior on perceived usefulness, change construct, and perceived ease of use

**Independent variable**	**Beta**	**T**	**p-value**	**R**^**2**^	**CI**
**PU**	0.327	2.84	0.006	0.383	19.95
**Change**	0.253	2.43	0.017	0.045	20.88
**PEU**	0.227	2.32	0.022	0.035	27.33
F = 23.4	Model R^2^				
p = 0.000	adj = 0.41				

SMR was also used to determine which particular items in each scale had a significant relationship to the overall acceptance of the PACS. The result of the SMR revealed that three variables (PACS enhancing effectiveness on the job (for PU), learning to use PACS has been easy (for PEU), and PACS has made the job more pleasant (for the change construct)) were significantly related to overall acceptance.

### Acceptance level and socio-demographic variables

Technologists had lower acceptance levels than did other professional groups (Table 5), but ANOVA results revealed that the overall differences in acceptance levels between the various radiology staff represented were not significant (F = 1.479 and p > 0.05), possibly because of the dominance of technologists in the sample. Table 5 also reveals that although the PACS acceptance level was low among those greater than fifty years old, ANOVA results showed no overall significant differences between the respondents due to age differences (F = 1.252 and p > 0.05). However, results showed a significant difference between groups according to how frequently they use PACS (F = 1.985 and p < 0.05). Respondents who always use the PACS in their work had higher levels of acceptance than those who are occasional or frequent users. The results of t-tests (Table 5) indicate that the PACS acceptance level was not influenced by gender or by the length of PACS experience.

**Table 5:** Differences in acceptance (behavioral construct) due to socio-demographic variables (t-test for variables with two groups, ANOVA for variables with more than two groups)

	**Variable**	**Mean**	**Std Dev.**	**Test value**	**p- value**
**Gender**	(1) Male	3.96	0.51	**0.291**	**0.59**
	(2) Female	3.73	0.54		
**Age**	(1) 22-30	3.77	0.51	**1.252**	**0.25**
	(2) 31-39	4.18	0.49		
	(3) 40-49	3.78	0.54		
	(4) ≥ 50	3.57	0.58		
**Job Title**	(1) Consultant	3.86	0.58	**1.479**	**0.13**
	(2) Radiologist	3.88	0.31		
	(3) Resident	4.05	0.41		
	(4) Technologist	3.02	0.54		
	(5) Other	4.50	0.24		
**PACS experience**	(1) ≤ one year	3.74	0.51	**0.384**	**0.54**
	(2) > one year	3.89	0.54		
**Use of the PACS**	(1) Always	3.88	0.53	**1.985**	0.024*
	(2) Frequently	3.41	0.28		
	(3) In the past but not now	3.44	0.0		

## Discussion

To date, only three TAM-related databases have been generated specifically for the study of PACS acceptance. The present study therefore adds to the small knowledge base of TAM-based PACS acceptance studies. Reviews of the TAM in healthcare-related studies (covering a variety of health technologies) show that the basic (original) model usually explains 30-40 % of IT acceptance (behavioral intention to use or behavior regarding new technology), and that its variations/extensions generally explain 40-60 % of physicians’ acceptance of new technologies [22]. The variance explained in the model here (41 %) is consistent with the range expressed in the literature.

### Factors influencing user acceptance

All three antecedent constructs—PU, PEU, and change—were found to be statistically significant predictors of IT user acceptance. PU was the most important individual predictor (38 % of variation explained), followed by the change scale (4.5 %) and PEU (3.5 %). In their overview of TAM studies in healthcare, Holden and Karsh [22] discovered 16 studies that had tested the relationship between PU and the outcome variable, all of which were significant. In contrast, 7 out of 13 studies that tested the influence of PEU found it to be significantly related to the outcome variable. The apparent lesser influence of PEU on user IT behavior found for PACS users at KAMC is therefore consistent with existing studies of acceptance of various technologies in healthcare settings. In this context, this finding has been explained in previous studies by the above-average intelligence of physicians and technologists [15].

Specifically regarding PACS technology acceptance, Duyck et al. [6] using the UTAUT model found that PU was a strong determinant of physicians’ and radiologists’ behavioral intention to use PACS (overall model R^2^ adj. of 33 %, 21 months post-implementation), but PEU was insignificant. Duyck et al. [23] also using UTAUT discovered that PU was the best predictor of acceptance, with PEU also being a significant predictor, and the overall model R^2^ adj. was 37 % (all PACS-using physicians but not radiologists, 21 months post-implementation). In a related study, Duyck et al. [26] achieved an R^2^ adj. of 48 % for the UTAUT studying radiologists and technologists. Pare et al. [24] used the Information Systems Success model, which differs from the TAM and its variants in both the suite of independent variables used and in the ultimate dependent variable. However, in their study, 24 months after PACS implementation, PU was a significant influence on “user satisfaction” for clinicians but not for radiologists or technologists, and PEU was significant for radiologists but not for technologists or clinicians. Pynoo et al. [25] used the UTAUT and path analysis (including indirect effects on acceptance via PU and PEU), and discovered that PU influenced physician acceptance at both 4 and 16 months post-implementation, whereas PEU was insignificant at both times (although PEU was found to be important at the actual time of implementation). The multiple correlation coefficient for acceptance was 0.60 at 16 months, meaning about 36 % of the variation in acceptance was explained by the overall model.

When the present findings regarding antecedents of acceptance are also considered, it would appear that PU is much more important than PEU for PACS acceptance. The percentage of variance in user acceptance explained by the various models used in PACS studies (including this investigation) lies in a fairly tight range of 33-48 %. Although this level of explanation is quite reasonable, it seems that there is a ceiling of explanation for PACS acceptance using TAMs (including variants/extensions). Some predictors evidently remain unknown or incompletely quantified given that more than half of the variance in acceptance remains unexplained. The change construct employed in this study showed some promise as a predictor of IT acceptance, and its use could be considered in future studies.

### Influence of professional user groups, gender, age, and experience

Duyck et al. [6], using the UTAUT, surveyed the acceptance of a PACS by radiologists and hospital physicians in a Belgian hospital. User acceptance ratings were higher for radiologists than for physicians, apparently because the former group experienced more benefits of the PACS and also had to use PACS more often through the working day. Duyck et al. [26] found that radiologists had higher ratings on PU, PEU, and acceptance than did technologists. However, Pare et al. [24] found similar acceptance ratings between radiologists, clinicians, and technologists. The present results show that there was no overall difference between professional user groups in PACS acceptance levels, although technologists had the lowest level of acceptance out of the five user groups surveyed. Technologists may have lower acceptance ratings as they are involved in PACS in a more limited way than are clinicians and radiologists [24], who additionally use the system for diagnostic and interventional purposes; alternatively, the training/familiarization program used (although extensive) may not have catered to their particular needs as successfully as it did for clinicians and radiologists.

Although various papers have found that age and gender are significant factors in attitudes toward IT in general, the review of Ward et al. [11] specifically on healthcare-related studies found no strong evidence for either an age or gender influence. Duyck et al. [6] found that male users rated PU more highly than did females, but it should be noted that in the UTAUT, gender is treated as a moderating influence and has no direct effect on acceptance. This study found there was no significant difference between men and women in PACS acceptance levels, or any influence of age, supporting the finding of Pare et al. [24].

Some healthcare studies have revealed positive relationships between IT acceptance and the years of experience in computer use [11]. Duyck at al. [23], using the UTAUT, discovered that the length of experience with PACS led to an overall improved attitude toward PACS. At KAMC, the length of experience using the technology had no direct influence on staff acceptance. This may reflect the particular professional groups surveyed, being radiologists and referring physicians in Duyck et al. [23] and additionally including technologists in this study, or it may be due to differences in the training/familiarization programs run at each hospital. The contrasting findings are unlikely to be due to PACS system architecture and features, as the hospitals in the respective studies both use General Electric PACSs. The very recent study of Pynoo et al. [25] found that although there was no main effect of experience on acceptance, it did show interaction effects with PU at 16 months post-implementation.

Overall, the findings in the literature (e.g. [11]) regarding professional grouping, gender, age, and experience with respect to acceptance of both PACSs and other health-related information technologies are mixed, and none of the characteristics appears to be a consistent indicator of users’ acceptance. This may partly reflect the differences between studies in the formulations of models used [15].

### Successful PACS adoption

There are many factors that influence whether an IT system, including a PACS, is successfully adopted into a healthcare organization, including organizational (managerial and structural) factors, technological factors, and behavioral factors [3,30]. User acceptance is a major factor influencing successful PACS adoption, as indicated by previous investigations (e.g., [19,21,27,30]). If the technology is not used, or under-used, or mis-used, then many of the tangible and intangible benefits of PACSs [1,2,4,5] relating to organizational efficiency, financial considerations, and improved patient care may not be fully realized. Resistance to IT among clinicians is well known ([3,10,30,31]), and such resistance has led in too many instances to failed, prolonged, or suboptimal implementation of new systems [10]. Resistance can be traced to various problems, including poor input into managing organizational change, insufficient pre-implementation training, installation issues, and personal-level factors including physicians’ time for adopting new technology, skepticism of the reliability and benefits of new technology, and users’ IT familiarity and ability [3,10,15]. PACS users at KAMC benefitted from a long (14-month) period of familiarization and training prior to the “go live” date. It is likely that this was an important facet of enhancing the subsequent levels of staff satisfaction and acceptance of the PACS, and hence was an important part of successful system adoption (e.g., [7]). Ayal and Seidmann [5], in their study of PACS implementation at a hospital in Rochester, New York, found that that physician satisfaction with the PACS service post-implementation was highly correlated with satisfaction with the implementation process, suggesting that sufficient and proper training is critical in increasing the level of users’ acceptance.

The two contrasting case studies of Pare and Trudel [3] exemplify the importance of the role of staff behavior and acceptance in an institution’s successful (or otherwise) adoption of a PACS. In respect of such resistance/acceptance, the findings of this study should be regarded as very encouraging. The overall level of acceptance for PACS users at KAMC is high, as indicated by the high mean values for the model constructs (PU 4.33/5, PEU 4.15/5, and acceptance 3.86/5). Previous studies of PACS acceptance also report positive attitudes from surveyed users. Duyck et al. [6] reported 86 % of responding physicians as giving positive scores (i.e. 4/7 or better) for PU, and 98 % for PACS acceptance. Pynoo et al. [25] recorded mean scores for PU, PEU, and acceptance of 4.70/7, 5.06/7, and 6.29/7, respectively, for physicians. The present study’s mean PU and PEU scores are higher (proportionally) than reported by Pynoo et al., but the acceptance score is lower. The lower acceptance score is probably due to the technologists surveyed here, who reported lower acceptance ratings than other user groups including radiologists and clinicians.

Cultural beliefs and values are considered by some researchers to play a part in the adoption, acceptance, and use of technology [32-34]. Straube et al. [34] refer to technical, organizational, and human constraints in Arab countries concerning IT transfer, which lead to resistance to technology. Based on arguments concerning culture-specific beliefs and values in Arab countries, it might be expected that the surveyed users would exhibit lower levels of PU and acceptance than their western counterparts, and that women would show lower levels than men. Although this study did not specifically test for a cultural influence, the high levels of PU and of user acceptance and the lack of consistent differences across different types of user (e.g., male v female) suggest that acceptance of PACS does not contain a cultural dimension in this case, supporting the result of Baker et al. [35] for knowledge workers in Saudi Arabia. The current finding may reflect the fact that the radiology department staff surveyed comprised a mix of cultures and nationalities, including Americans, Australians, and Europeans, as well as Saudi nationals: the increasing global mobility of healthcare professionals may reduce/remove the influence of host cultural elements in user acceptance of PACSs, as well as that of other health IT systems. The finding may also be due to the PACS being supplied by a multinational vendor (GE), rather than by a local vendor.

## Conclusions

User acceptance is a critical factor in the success of healthcare IT adoptions. Given the paucity of investigations into users’ acceptance of PACSs, and the absence of such studies in Arab countries, a variant of the TAM was applied to survey data of radiology department staff regarding the PACS installed at KAMC hospital in Riyadh, Saudi Arabia. The results reveal high levels of perceived usefulness (PU), perceived ease of use (PEU), and user acceptance. PU, PEU, and a change construct all have a significant effect on radiology staff acceptance, although PU is by far the most influential determinant. The most important item in the PU construct was that users found the PACS to have improved the quality of their work in providing better patient care. Regarding PEU, the vast majority of the staff agreed that learning to use the PACS has been easy, and with respect to the change construct, that the system has made their job more interesting, pleasant, and less stressful. Forty-one percent of the variation in PACS acceptance at KAMC was explained by the determining variables, which suggests that other factors are likely influencing acceptance besides those measured.

User acceptance of PACS technology must be maximized in any host healthcare organization if the tangible and intangible benefits of the systems are to be realized. The recommendations of the study therefore include: (i) The quality of the PACS system and the ability to accomplish tasks more quickly should be considered as important items in PACS implementation; (ii) Health organizations should look for a PACS that offers all required functions and which is likely to engender high user PU, rather than a system that is easy to use, as usefulness is much more important in determining staff acceptance; (iii) Training and familiarization programs for PACS implementation should focus on establishing high levels of PU in the users. Training programs should be tailored to different professional groups, particularly technologists, who had lower levels of acceptance than other groups; and (iv) Health organizations should attempt to measure the factors that influence the acceptance of a PACS by their staff, in order to optimize the productivity of the system and realize the potential benefits to the greatest possible extent.

Future research studies should investigate several issues regarding PACS acceptance. First, the lower acceptance levels indicated by technologists compared with physicians and radiologists should be examined more closely, including identifying the likely determinants (e.g., their frequency of PACS use, their perceived benefits of the system, and the sufficiency, content, and emphasis of training). Second, a future study could examine cultural effects more explicitly, including any influence of PACS user nationality and of whether the supplier of the system and training program is local or multinational. About half of the PACSs in the Middle-East are supplied by local IT companies and the other half by multinationals [13]; differences may exist between the two groups of suppliers regarding not only the systems, but also the implementation process and training programs provided. Third, other potential predictors of user acceptance need to be found, given both the importance of acceptance in successful PACS adoption and the moderate (<50 %) explanation levels of variance in acceptance currently afforded by existing models.

## Competing interests

The author declares that he has no competing interests.

## Pre-publication history

The pre-publication history for this paper can be accessed here:

http://www.biomedcentral.com/1472-6947/12/44/prepub
